# The Correlation Between Lipid Profile and Stress Levels in Central Iran: Isfahan Healthy Heart Program

**Published:** 2010

**Authors:** Maryam Shahnam, Hamidreza Roohafza, Masoumeh Sadeghi, Ahmad Bahonar, Nizal Sarrafzadegan

**Affiliations:** 1 Research assistant, Isfahan University of Medical Sciences, Isfahan, Iran; 2 Psychiatrist, Isfahan Cardiovascular Research Centre, Isfahan University of Medical Sciences, Isfahan, Iran; 3 Associated Professor of Cardiology, Isfahan Cardiovascular Research Center, Isfahan University of Medical Sciences, Isfahan, Iran; 4 Research assistant, Isfahan Cardiovascular Research Center, Isfahan University of Medical Sciences, Isfahan, Iran; 5 Professor of Cardiology, Isfahan Cardiovascular Research Center, Isfahan University of Medical Sciences, Isfahan, Iran

**Keywords:** Cholesterol, Triglycerides, Stress, Adult

## Abstract

**BACKGROUND:**

Previous studies suggest that mental status may influence serum lipid levels. This study was conducted on adult population living in rural and urban areas in Central Iran to assess the correlation between stress level and lipid profile disorders.

**METHODS:**

Data was extracted from final evaluation of Isfahan Healthy Heart Program (IHHP) in 2008. Multistage and random cluster methods were used for sampling. The study population consisted of 9752 adults aged ≥19 years living in three districts namely Isfahan, Arak and Najaf Abad. Demographic data, age and sex were recorded. Blood samples were taken to determine the lipid levels including total cholesterol (TC), low-density lipoprotein cholesterol (LDL-C), low levels of high-density lipoprotein cholesterol (HDL-C) and triglycerides. Stress levels were assessed using the General Health Questionnaire. Logistic regression and chi-square tests were used for statistical analysis.

**RESULTS:**

The odds ratios of high stress in individuals with high levels of TC, LDL-C and low levels of HDL-C compared to normal individuals after adjustment for age and sex were as follows respectively: 1.05 (1.02,1.15), 1.06 (1.02,1.18), 1.06 (1.01,1.17).

**CONCLUSION:**

Intervention activities towards reduction of stress levels at the community level may be useful as part of the strategy for cardiovascular disease prevention.

## Introduction

Cardiovascular diseases (CVD) are recognized as important threats to human health.^1^ It is the leading cause of death in Iran, as well.^2^ Blood lipids are influenced by nutrition, body weight, physical activity, medications and genetic factors.^3^, ^4^ Evidence suggests that blood lipids are also affected by mental status.^5^ It is postulated that stress increases blood lipids through increasing hepatic lipoprotein lipase activity caused by a heightened sympathetic neuronal response.^6^ Hence stress may have a role in causing CVD. Various studies have investigated the influence of mental factors on the levels of different blood lipids. A review was done by Dimsdale suggests that the levels of free fatty acids and total cholesterol rise following acute and/or chronic stress.^7^ Another study conducted in 2007 in Tabriz, Iran, demonstrated an increase in serum triglycerides in individuals exposed to stress in the past 6-12 months.^8^ Few studies have investigated a possible link between high density lipoprotein cholesterol (HLD-C), low density lipoprotein cholesterol (LDL-C) levels and stress.^9^, ^10^ According to Isfahan Healthy Heart Program (IHHP) a comprehensive community based program for CVD prevention and control, more than half of the population in Central Iran had some disturbance of lipid profile.^11^ We aimed to assess the possible correlation between lipoproteins disorders and stress level in adult population of Central Iran.

## Materials and Methods

The current study is based on the data obtained from the final evaluation of IHHP in 2007. Details of IHHP, including sample size, data entry and data analysis were published previously.^12^ Sampling was performed using multistage and random cluster method. The study population in the districts of Isfahan, Najaf Abad, and Arak were classified into urban and rural based on 2006 public census.

Nearly 5–10% of the population was enrolled in the study. Individuals’ aged ≥ 19 years were randomly selected from each house. The calculated sample size was 9572 people. Sample size in this study was calculated according to age, sex and place of residence. These three districts have less varied population with fewer immigrants compared to Iran's capital and other cities. The participants had to have lived in these regions for at least 10 years. The exclusion criteria were pregnancy, mental retardation. Informed written consent was obtained from all participants after they were briefed about the study. Other information was obtained in house visits by trained personnel. The next day, blood samples were taken from these individuals after 12 hours of fasting to measure triglyceride (TG), total cholesterol (TC), LDL-C and HDL-C. TC and HDL-C measurements were conducted via using a Toshiba auto analyzer. LDL-C was calculated with Friedewald's formula.^13^ HDL-C was measured using the calorimetric enzymatic method.

Demographic factors included age (19–24, 25–34, 35–44, 45–54, >50 years), sex (male, female), marital status (married, single) education (0-5, 6-12, >12 years) and residence area (urban, rural).

### Definitions

TC ≥240 mg/dl and/or receiving cholesterol-lowering medications were considered hypercholesterolemia. Fasting TG ≥200 mg and/or receiving TG-lowering medications were considered hypertriglyceridemia. LDL-C ≥160 mg/dl, HDL-C ≤40 mg/dl in men and HDL-C ≤ 50 mg/dl in women were considered abnormal levels.^14^, ^15^ To evaluate stress, the General Health Questionnaire 12 (GHQ12) self-administered questionnaire for general health assessment, was used. This questionnaire consists of twelve 4-choice questions. To determine each individual's stress score based on the GHQ scoring system, choices (a) or (b) were given a score of 0 and for choices (c) and (d) a score of 1 was considered. A total score ≥4 was considered high GHQ (i.e. high stress).^16^ This questionnaire has been designed for individuals aged 12 years and higher and its validity/reliability has been studied and previously reported in Iran.^17^

### Statistical Analysis

Data were analyzed using SPSS 15. P<0.05 was considered statistically significant. Age groups, sex, residence area, education and lipid profile disorders were expressed in percentages and analyzed using chi-square test. Logistic regression model was used to assess the correlation between stress level and lipid profile. Independent variables, include hypercholesterolemia, hypertriglyceridemia, increased LDL-C, and decreased HDL-C. Age groups,sex and stress levels were considered dependent variables.

## Results

High stress levels (GHQ ≥4) were observed in 39.6% of women and 28.8% of men. 34.6% of city dwellers and 38.5% of non-married individuals demonstrated high stress levels. Among individuals with 0-5, 6-12 and more than 12 years of education, the percentage of those with GHQ ≥4 was 37.7%, 32.1% and 28.7%, respectively. Other demographic characteristics of the study population are presented in Table 1.

**Table 1:** Demographic characteristics of the study population according to stress level: IHPP

		Low stress	High stress	OR	95% CI
Age(years)	19–24 years	1241(66%)	641(34%)	0.84	(0.73, 0.96)
	25–34 years	1952(67.1%)	959(32.9%)	0.80	(0.70, 0.90)
	35–44 years	1269(67.1%)	622(32.9%)	00.79	(0.69, 0.91)
	45–54 years	778(66.4%)	6393(33.6%)	0.82	(0.69, 0.91)
	≤55 years	1046(61.9%)	645(38.1%)	1	Ref
Sex	Female	2887(60.4%)	1889(36.9%)	1.62	(1.49, 1.76)
	Male	3402(71.2%)	1375(28.8%)	1	Ref
Residency area	Rural	1924(66.9%)	953(33.1%)	1.07	(0.97, 1.17)
	Urban	4365(65.4	2311(34.6%)	1	Ref
Marital status	Single	1310(61.5%)	621(38.5%)	1.28	(1.15, 1.41)
	Married	4977(67.1%)	2440(32.9%)	1	Ref
Education (years)	0–5 years	2677(62.3%)	1619(37.7%)	1.50	(1.31, 1.72)
	6–12year	2699(67.9%)	1278(32.1%)	1.17	(1.02, 1.35)
	> 12 year	901(71.3%)	363(26.7%)	1	Ref

Table 2 refers to the Odds ratio (OR) of high stress in hypercholesterolemic individuals is 1.24 times as high as those with normal TC. The OR of high stress in individuals with LDL-C ≥160 mg/dl was 1.21times as high as in those with low LDL-C. In women and men studied, the OR of high stress in those with HDL-C was 1.32 times as high as individuals with normal HDL-C.

**Table 2:** Prevalence of dyslipidemia associated with stress level in the studied population: IHPP- logistic regression analysis.

Lipid profile		High stress	Low stress	OR	95% CI
TG (mg/dl)	< 200	595(32.4%)	1242(67.6%)	0.89	(0.82,1.09)
	≥200	2630(34.6%)	4964(65.4%)	1	Ref
TC(mg/dl)	<240>	435(36.4%)	760(63.6%)	1.24	(1.17,1.45)
	≥240	2789(33.9%)	5447(66.1%)	1	Ref
LDL-C (mg/dl)	<160	384(36.6%)	666(62.4%)	1.21	(1.12,1.38)
	≥160	2840(33.9%)	5538(66.1%)	1	Ref
HDL-C (mg/dl	Male <40	1807(35.5%)	3285(64.5%)	1.32	(1.11,1.48)
	Female <50				
	Male ≥40	14.9(32.6%)	2911(67.4%)	1	Ref
	Female ≥50				

TG, triglyceride; TC, Total cholesterol; LDL-C, low-density lipoprotein cholesterol; HDL-C, high-density lipoprotein cholesterol

No significant relationship was found between high stress level and increased blood TG.

In the multivariate logistic regression model, the Odds ratio of high stress in high TC, high LDL-C, and low HDL-C compared to normal levels was 1.11, 1.13, and 1.12 times higher, respectively.It remained significant after age and sex adjustment (table 3).

**Table 3:** Crude and Adjusted Odds ratios of blood lipids associated with stress levels: IHPP

Lipid profile	Unadjusted OR( 95% CI)	Adjusted (sex and age) OR( 95% CI)
TG (mg/dl)	0.90(0.81,1.00)	0.92(0.82, 1.03)
TC(mg/dl)	1.11(1.08, 1.27)	1.05(1.02, 1.15)
LDL-C (mg/dl)	1.13(1.04, 1.24)	1.06(1.02, 1.18)
HDL-C (mg/dl)	1.12(1.08, 1.28)	1.02(1.01, 1.17)

TG, triglyceride; TC, Total cholesterol; LDL-C, low-density lipoprotein cholesterol; HDL-C, high-density lipoprotein cholesterol

## Discussion

We investigated the correlation between stress level and lipid profile. Based on our results, the Odds ratio of high stress in high TC, high LDL-C and low HDL-C was 1.11, 1.13 and 1.12 times higher compared to normal individuals.

The findings of this study are consistent with previous studies on the relationship between stress level, high TC and LDL-C. Patterson and colleagues studied the effect of mental stress on lipid profile in 1993. The study showed that stress increases TC and LDL-C.^5^ The study of Bacon and colleagues in 2004 demonstrated that stress increased TC and LDL-C levels in 51 patients with suspected coronary artery disease.^18^ Another study conducted in 2008 among 20627 individuals investigating the relationship between mental stress and cardiovascular risk factors demonstrated high stress levels in individuals with abnormal cholesterol levels.^19^ A review reported by Dimsdale and Herd showed that the level of free fatty acids and TC increase in acute and chronic stress.^7^

While a study conducted by Fakhari and colleagues in 2004 demonstrated increased TG in individuals who had experienced high levels of stress in the preceding 6–12 months.^8^ Our study did not show any relationship between stress and high TG. In regard to the differences between these findings, as well as the effect of other factors such as obesity, diabetes, medications, alcohol and genetics in increasing TG, further studies on the possible correlation between stress and high TG are warranted.

Although in this study, individuals with low HDL-C had higher levels of stress compared to normal individuals, some earlier studies have reported an association between high stress and increased HDL-C.^5^, ^6^

Studies concerned with explaining the pathophysiological mechanisms underlying lipid profile disorders and mental stress emphasize that following acute stress, serum cholesterol concentration increases. Activation of the sympathetic nervous system in stressful episodes increases free fatty acids. On the other hand, chronic stress is associated with behaviours such as intake of high-fat meals, cigarette smoking and drinking alcohol, which in turn lead to disorders of lipid profile.^6^

## Conclusion

Our results demonstrated high stress levels in individuals with high TC, high LDL-C, and low HDL-C compared to individuals with normal lipid profile. Considering the importance of prevention in dealing with cardiovascular diseases, it seems that improving intervention strategies for stress reduction in communities may be a beneficial strategy in preventing cardiovascular diseases.
